# A New Family of Intrinsically Disordered Proteins: Structural Characterization of the Major Phasin PhaF from *Pseudomonas putida* KT2440

**DOI:** 10.1371/journal.pone.0056904

**Published:** 2013-02-15

**Authors:** Beatriz Maestro, Beatriz Galán, Carlos Alfonso, Germán Rivas, Maria A. Prieto, Jesús M. Sanz

**Affiliations:** 1 Instituto de Biología Molecular y Celular, Universidad Miguel Hernández, Elche, Spain; 2 Centro de Investigaciones Biológicas, Consejo Superior de Investigaciones Científicas, Madrid, Spain; University of South Florida College of Medicine, United States of America

## Abstract

Phasins are intracellular polyhydroxyalkanoat4e (PHA)-associated proteins involved in the stabilization of these bacterial carbon storage granules. Despite its importance in PHA metabolism and regulation, only few reports have focused so far on the structure of these proteins. In this work we have investigated the structure and stability of the PhaF phasin from *Pseudomonas putida* KT2440, a protein that is involved in PHA granule stabilization and distribution to daughter cells upon cell division. A structural, three-dimensional model of the protein was built from homology modeling procedures and consensus secondary structure predictions. The model predicts that PhaF is an elongated protein, with a long, amphipathic N-terminal helix with PHA binding capacity, followed by a short leucine zipper involved in protein oligomerization and a superhelical C-terminal domain wrapped around the chromosomal DNA. Hydrodynamic, spectroscopical and thermodynamic experiments validated the model and confirmed both that free PhaF is a tetramer in solution and that most part of the protein is intrinsically disordered in the absence of its ligands. The results lay a molecular basis for the explanation of the biological role of PhaF and, along with an exhaustive analysis of phasin sequence databases, suggest that intrinsic disorder and oligomerization through coiled-coils may be a widespread mechanism among these proteins.

## Introduction

Natural polyhydroxyalkanoates (PHAs) are organic polyoxoesters composed of (R)-3-hydroxy fatty acids which constitute the carbon and energy storage material of certain bacterial species under nutrient limitation conditions [1]–[6]. PHA is synthetized in the cytoplasm of bacteria and accumulates as multiple granules composed by the polyester (93–97% of the cell dried weight, CDW) coated by a phospholipid monolayer (1–6% of CDW) and proteins associated to the granule (1–2% of CDW), forming a layer at the granule surface [3]. Currently four classes of proteins associated to the bioplastic granules (GAP) have been defined: *i*) the PHA synthases, involved in the PHA polymerization, *ii*) the PHA depolymerases, implicated in bioplastic degradation and monomer mobilization, *iii*) the so-called phasins, devoided of enzymatic activity, and *iv*) other proteins [3], [7].

PHAs are considered enviromental-friendly plastics because they are biodegradable and biocompatible materials, so they have attracted much attention in diverse fields such as biotechnology, biomedicine, and agriculture [8]–[11]. Therefore, it follows that the understanding of protein-PHA interactions from a biophysical point of view will undoubtedly widen the biotechnological and clinical applications of these bioplastics. In this sense, phasins are low molecular weight proteins that constitute the major GAP fraction [12] and have been identified in different bacteria such as *Ralstonia eutropha* [13], *Bacillus megaterium* [14], *Rhodococcus ruber* [15], *Paracoccus denitrificans* [16], *Pseudomonas putida* [17]–[19], and *Acinetobacter sp* [20]. These proteins are only synthesized when the PHA is produced [13]; [21]–[23] and play an important role in stabilizing the PHA granules, preventing them from coalescing and affecting their size and number in the cells [3], [18], [24]. Moreover, phasins are involved in the structure and in the degradation of poly(3-hydroxybutyrate) granules [25]. Besides their structural roles, phasins play also a regulating role in PHA metabolism. In this sense, the phasin PhaP from *Ralstonia eutropha* increases the specific activity of type II polyhydroxyalkanoate (PHA) synthases PhaC1 and PhaC2 from *Pseudomonas aeruginosa* [26]. We have recently demonstrated how PhaF, the major phasin from *P. putida* KT2440, is involved in the control of expresion of the *pha*C1 synthase and *pha* I phasin genes, as well as in granule localization within the cell and granule segregation during cell division [18].

In spite of their extensive physiological characterization, little is known about the structure and folding of phasins. From the analysis of the amino acid sequence we have previously suggested that the PhaF phasin from *P. putida* is structured in two domains: *i*) the N-terminal domain, with a possible function of binding to the PHA granule, and *ii*) the C-terminal domain showing characteristic 4- and 5-aa repetitions similar to those found in DNA-binding, nucleoid-associated proteins [17], [18]. Several evidences support the structural and functional independence of the domains. First, the PhaI phasin, which shares considerable sequence similarity with the N-terminal region of PhaF (57% similarity, 38% identity) [17] is capable of acquiring a folded, stable and functional structure by itself [27]. Also, the N-terminal domain is conserved in other PHA-binding phasins with a different C-terminal part [17], [18]. Moreover, very diverse fusion proteins containing the N-terminal moiety of PhaF (the BioF affinity tag) can also be adsorbed on polyhydroxyalkanoate granules without compromising their function [27], [28]. Finally, the isolated C-terminal moiety is able to bind to DNA [18].

The importance in understanding the molecular basis underlying the PHA-phasin interaction and its biological consequences, together with its biotechnological application such as the BioF tag, prompted us to characterize the structure and stability of the PhaF phasin of *P. putida* KT2440 by ultracentrifugation and optical spectroscopy techniques, and to ellaborate a model of its three-dimensional structure explaining the binding to both PHA granules and DNA. The results suggest a peculiar structural organization of PhaF that supply an explanation to its biological role as coupling agents between PHA and the bacterial genetic material, and establish the basis for the designing of new variants of these proteins with novel biochemical properties.

## Materials and Methods

### Chemicals

Urea, ammonium sulphate, carboxymethyl cellulose, bovine pancreas deoxyribonuclease, magnesium chloride, sodium oleate, 2,2,2-trifluoroethanol and isopropyl β-D-1-thiogalactopyranoside (IPTG) were purchased from Sigma-Aldrich (St. Louis, MO, USA). Butyl-sepharose-4 Fast Flow was from General Electric Healthcare (Piscataway, NJ, USA).

### Molecular modeling

**Figure 1:**
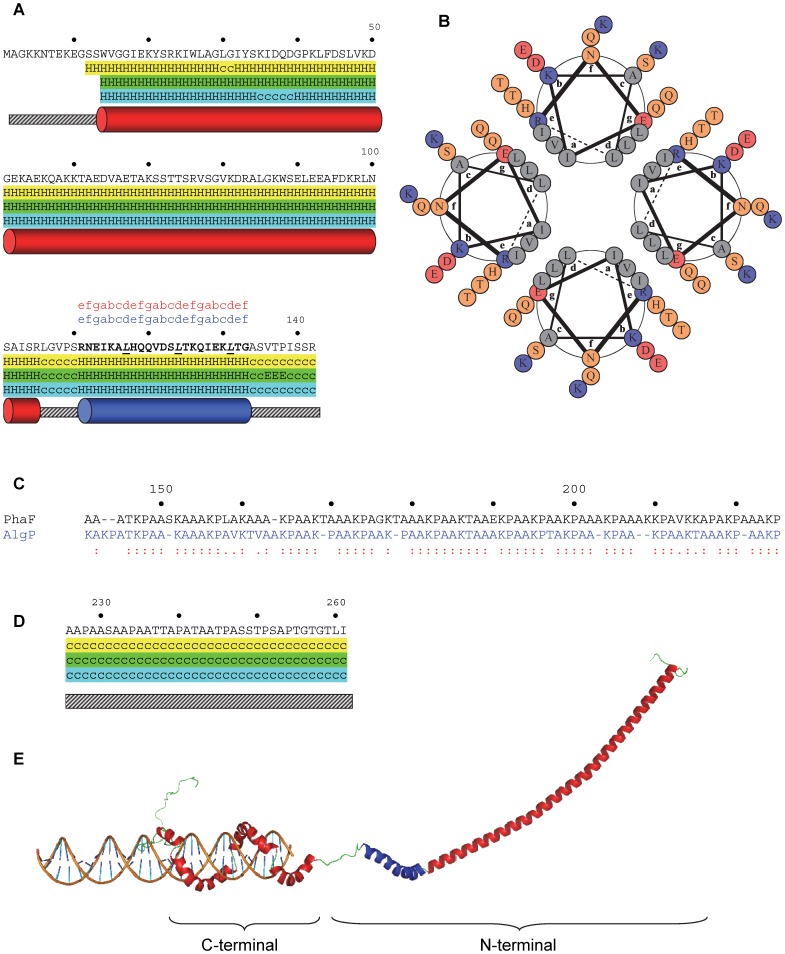
Three structural model of the PhaF phasin. (**A**) Secondary structure prediction of the N-terminal domain carried out by the PHD (yellow), Jpred (green) and MLRC (cyan) methods. *H*, alpha-helix; *E*, extended (beta); *c*, random-coil. Consensus helices are represented by cylinders. Boldface residues constitute the predicted coiled-coil stretch by the Lupas (red) and MARCOIL (blue) methods, indicating the scheme of the heptad repeats above the sequence. Leucine residues within the coiled-coil are shown underlined and italicized. (**B**) Helical-wheel representation of the predicted leucine zippers interacting as a parallel tetramer, using DRAWCOIL. (**C**) Sequence comparison between PhaF (black) and AlgP (blue). Colons represent identical amino acids, and dots indicate conservative changes. (**D**) Secondary structure prediction of the C-terminal tail. Color scheme as in (A). (**E**) Joint model structure of monomeric PhaF complexed with DNA. The leucine zipper is shown in blue.

The three-dimensional structure of the N- and C-terminal domains of PhaF protein was modeled separately as follows:

- *N-terminal domain (residues 1-142) and C-terminal tail (residues 226-261)*. Secondary structure prediction was carried out by three different methods, *i.e*. PredictProtein (PHD, http://www.predictprotein.org) [29], JPred (http://www.compbio.dundee.ac.uk/www-jpred/) [30] and multivariate linear regression combination (MLRC) [31] (http://npsa-pbil.ibcp.fr/cgi-bin/npsa_automat.pl?page=/NPSA/npsa_server.html) (Fig 1A). A consensus prediction was performed by assigning a particular secondary structure to each residue if that was predicted by at least two of the methods. Helical coiled-coil sequences were predicted by the method of Lupas *et al*. [32] in the COILS server (http://www.ch.embnet.org/software/COILS_form.html) and MARCOIL (http://bcf.isb-sib.ch/webmarcoil/webmarcoilC1.html) [33], selecting in both cases the segments with coil probability above 75%. The helical wheel representation of the coiled-coils was accomplished with DrawCoil 1.0 (http://www.gevorggrigoryan.com/drawcoil/). To build the three dimensional model, the coiled-coil predicted stretch (residues 111-133) was modelled using Swiss PDB Viewer 3.7 [34] using tropomyosin, a known coiled-coil protein, as the template (Protein Data Bank -PDB- code 1C1G). The rest of the domain was also modelled using the Swiss PDB Viewer 3.7 utility, assigning standard α-helix (φ = −65°, ψ = −40°) or randomized Ramachandran angles taking into account the consensus prediction.
- *C-terminal domain (residues 143-225)*. This section was *de novo* modeled using as a guide the theoretical model of the DNA-binding region of the AlgP protein from *Pseudomonas aeruginosa* [35]. This model assigned standard helix and coil Ramachandran angles to the different 4- and 5-aa repeats found in AlgP. A sequence alignment allowed the identification of these repeats in the C-terminal domain of PhaF (Fig 1C). Therefore, we assigned the same group of standard angles to those repeats that were conserved between the two proteins.
- *Overall structure*. The peptidic backbones of the models for the N- and C-terminal domains were linked together using SwissPDB Viewer, and the whole structure was subjected to steepest descent energy minimization and checked for structural errors. In order to get a representation of the binding of PhaF to DNA, a three-dimensional model of the 31-bp oligonucleotide that was already shown experimentally to bind to PhaF [18] (5′-AATTCACAGTAAAACCAGATGGCTTGATTAC-3′, and its complementary strand) was generated with the *Model.it* server (http://hydra.icgeb.trieste.it/dna/model_it.html) and manually docked onto the PhaF model using Swiss PDB Viewer for presentation purposes (Fig. 1E). Figures were rendered with PyMol (Delano Scientific LLC).

Prediction of protein disorder was accomplished using the following methods, all contained in the Disprot Database of Protein Disorder (http://www.disprot.org/predictors.php): PONDR-FIT™ [36]; DISOPRED2 [37], selecting a false positive rate threshold of 2%; DisEMBL™ [38], selecting the predicted regions with nonassigned electron densities in the Protein Data Base (REM465); and DISpro [39].

### Protein purification

Full-length PhaF and its C-terminal domain (C-PhaF) were expressed, purified and quantified as previously described [18].

### Analytical ultracentrifugation

Sedimentation velocity experiments of were carried out by spinning a PhaF solution (38 µM; equilibrated in 20 mM sodium phosphate buffer, pH 7.0, plus 150 mM NaCl) at 48000 rpm and 20°C in an XL-A analytical ultracentrifuge (Beckman-Coulter Inc.) with UV-VIS optics detection system, using an An50Ti rotor and 12 mm double-sector centrepieces. Sedimentation profiles were registered every 5 minutes at 260 and 275 nm. The sedimentation coefficient distributions were calculated by least-squares boundary modelling of sedimentation velocity data using the c(s) method [40], as implemented in the SEDFIT program. These s-values were corrected to standard conditions (water, 20 °C, and infinite dilution) [41] using the SEDNTERP program [42] to get the corresponding standard s-values (s_20,w_). A sedimentation equilibrium experiment was carried out to determine the state of association of PhaF. Short-column (80–100 µl) equilibrium runs were carried out at 10000 and 16000 rpm) and the corresponding scans were measured at 238 nm, using the same experimental conditions and instrument as in the sedimentation velocity experiments. After the equilibrium scans a high-speed centrifugation run (43000 rpm) was done to estimate the corresponding baseline offsets. Weight-average buoyant molecular weight of the protein was determined by fitting a single species model to the experimental data using a MATLAB program (kindly provided by Dr. Allen Minton, NIH) based on the conservation of signal algorithm [43]. The corresponding protein molecular weight was determined from the experimental buoyant mass using 0.744 cm^3^/g as the partial specific volume of PhaF (calculated from the amino acid composition using the SEDNTERP program, [42]).

### Circular dichroism spectroscopy

Circular dichroism (CD) experiments were carried out in a Jasco J-810 spectropolarimeter equipped with a Peltier PTC-423S system. Isothermal wavelength spectra were acquired at a scan speed of 50 nm/min with a response time of 2 seconds and averaged over at least 6 scans at 20 °C. Protein concentration was 3.8 or 38 µM and the cuvette pathlength was 0.1 cm (far-UV) or 1 cm (near-UV). Molar ellipticities ([*θ*]) are expressed in units of deg cm^2^ (dmol of residues)^–1^. With urea present, spectra could not be recorded below 215 nm due to the high absorbance of the sample. Estimations of secondary structure content were calculated by deconvolution of the far-UV CD spectra using the CDNN program (Gerald Böhm, Institut für Biotechnologie, Martin-Luther-Universität Halle-Wittenberg, Germany) as well as the CONTIN [44] and CDSSTR [45] procedures contained in the Dichroweb utilities (http://dichroweb.cryst.bbk.ac.uk/html/home.shtml) [46], [47]. Buffer was 20 mM sodium phosphate, pH 7.0, unless otherwise stated. C-PhaF-DNA binding experiments were carried out as previously described [18]. Briefly, an unspecific DNA fragment (nspDNA) was prepared from hybridization of oligonucleotides 5′-AATTCACAGTAAAACCAGATGGCTTGATTAC-3′ and its complementary strand (Invitrogen), to a final stock concentration of 0.5 mg ml-1. The C-PhaF and nspDNA concentration was 9 mM. For the pH stability experiments buffers were 50 mM sodium phosphate (pH 6.0–8.0), 50 mM sodium acetate (pH 3.5–5.5) or 50 mM glycine (pH 2.0–3.0 and 9.0–9.5). Final pH was measured *in situ* using a Crison Basic-20 pH-meter. Samples were centrifuged 5 min prior CD measuring.

### Fluorescence spectroscopy

Emission scans were performed at 20 °C on an PTI-QuantaMaster spectrofluorimeter (Birmingham, NJ, USA), model QM-62003SE, using a 5×5 mm path length cuvette and a protein concentration of 3.8 µM. Tryptophan emission spectra were obtained using an excitation wavelength of 280 nm, with excitation and emission slits of 0.5 nm and 0.7 nm respectively, and a scan rate of 60 nm min^–1^. Wavelength of the maximum intensity was calculated by first derivative analysis. The average emission intensity, <*λ*>, in fluorescence spectra was calculated as depicted in Eq. 1
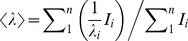
(1)where *I*_i_ is the fluorescence intensity measured at any *λ*_i_ wavelength. Buffers used were the same as for CD.

### Equilibrium denaturation

CD-monitored thermal denaturation experiments were performed in a 0.1 cm (far-UV) or 1 cm (near-UV) path cell. The sample was layered with mineral oil to avoid evaporation, and the heating rate was set to 1 °C min^−1^ unless otherwise stated. The thermodynamic analysis assumed a two-state unfolding-dissociation coupled mechanism of tetramer denaturation: 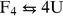
(2)


where F and U denote the folded and unfolded species, respectively. Effective free energies (Δ*G* ^o^ *_eff_*) were calculated from the CD titration traces:
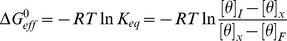
(3)where *K*_eq_ is the equilibrium constant between the initial and final states, [*θ*]_I_ and [*θ*]_F_ are the ellipticities of the initial and final state, respectively, and [*θ*]_x_ is the experimental ellipticity at a given urea concentration.

Thermal scans were fitted by least squares to the Gibbs-Helmholtz equation (Eq. 4): 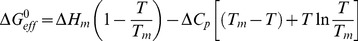
(4)where Δ*G* ^o^ *_eff_* (*T*) is the effective free energy of the transition, Δ*H*_m_ is the van't Hoff enthalpy, *T*_m_ is the midpoint of denaturation (in Kelvin) and Δ*C*_p_ is the difference in heat capacity between the native and denatured states. The intrinsic free energy of the tetramer (Δ*G* ^o^ *_int_*) is therefore calculated as follows:
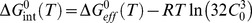
(5)being *C*_o_ the total monomer concentration. Reader is referred to Backmann *et al*. [48] for the detailed description of equations 4–5.

For urea titrations, aliquots from an 8.0 M denaturant stock solution containing the same concentration of protein as in the cuvette (to keep the protein concentration constant throughout the titration, this is, 3.8 µM) were added stepwise and incubated for 5 min prior to record the wavelength spectra. Experiments were performed at 10 °C in 20 mM sodium phosphate buffer, pH 7.0. Intrinsic free energy calculations (Δ*G* ^o^ *_int_*) were accomplished using eqs. 3 and 5, but in this case Δ*G* ^o^ *_eff_* was obtained using the linear extrapolation method [49]:
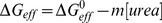
(6)where *m* denotes the dependence of Δ*G_eff_* with [urea], and Δ*G^o^_eff_* is the free energy in the absence of denaturant, which can be calculated as:
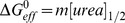
(7)being [urea]_½_ the midpoint of the transition.

## Results

### Three-dimensional structural model of PhaF

The setup of an efficient overexpression and purification system for PhaF [18] allowed the availability of high amounts of pure protein (Fig. S1) that was subsequently subjected to crystallization trials aimed to the elucidation of its structure by X-ray diffraction techniques. However, no suitable crystals have been obtained so far, either in the presence or absence of DNA, prompting us to elaborate instead a three-structural model of the protein. Homology modeling constitutes a commonly used procedure, provided that there are sufficiently close homologous sequences in the databases of protein structures. Nevertheless, only the C-terminal, DNA-binding domain displays some similarity with nucleoid-associated-like proteins [18]. Therefore, we decided to employ different procedures separately for each of the N- and C-terminal domains and then join together the two models into a single structure. As it will be discussed below, the predicted extended structure and lack of tertiary contacts between domains supported this approach.

The secondary structure of the N-terminal domain was predicted by three different methods as predominantely α-helical with a high consensus degree (Fig. 1A). A long, uninterrupted α-helix would span residues 13–105, followed by a shorter helix encompassing residues 111–133. Such a long helix is likely to be unstable unless it is involved in stabilizing protein-ligand or protein-protein interactions. It should be reminded that secondary structure prediction methods usually unveil local propensities of peptide segments which might not acquire such structure when isolated from the rest of the protein or other stabilizing ligands, the PHA granule in this case. The rest of positions (residues 1-12, 106-110 and 134-142) were mostly assigned random-coil conformations by the methods. Remarkably, this second helical stretch is also compatible with a heptad-repeat pattern characteristic of a helical coiled-coil conformation, as suggested from the analyses by the Lupas and Marcoil methods (Fig. 1A). The fact that all "d" positions are occupied by leucine residues strongly suggests that amino acids 111–133 conform a leucine-zipper involved in PhaF oligomerization (Fig. 1B). Hence, we assigned standard α-helical angles to the long helix, and tropomyosin-based coiled-coil angles to the short helix. Scarcity of irregular structures, that accumulate mostly at the extremes of the sequence, as well as of loop sequences, makes the N-terminal domain very likely to adopt an extended structure without loops and bends that allow significative tertiary contacts (Fig. 1E). One of the most remarkable features in the long helix is the segregation of polar residues from the hydrophobic ones on both sides of the helix, creating an amphipathic sequence in which the polar face is in turn very abundant in acidic and basic charged residues (Fig. S2).

With respect to the C-terminal domain, its highly repetitive sequence between residues 143–225 displays an appreciable similarity with that of the AlgP transcriptional regulatory protein from *P. aeruginosa* (Fig. 1C), whose structure was previously modelled [35]. This allowed the assignation of the Ramachandran angles to conserved residues, with very few gaps that were otherwise assigned random-coil values. Fig. 1E displays the final modelled structure of the C-terminal domain, within the entire protein, forming a superhelix able to be inserted into the major groove of DNA. Fig. S2 also shows the disposition of repeated lysine residues, suitably placed to interact with the negatively charged sugar-phosphate backbone in the DNA. The highly charged nature of this region suggests that electrostatic repulsions render it structurally disordered unless complexed with the nucleic acid.

Finally, residues 226–261 constitute a degenerated tail sequence derived in part from the DNA-binding moiety, but lacking any basic residues. No homologous sequences were found in the databases of proteins with known tertiary structure, and all methods predicted inequivocally a random-coil conformation, although this sequence contains some PXXP repetitions that are have been described to promote poly(proline)-II conformations (see below).

**Figure 2:**
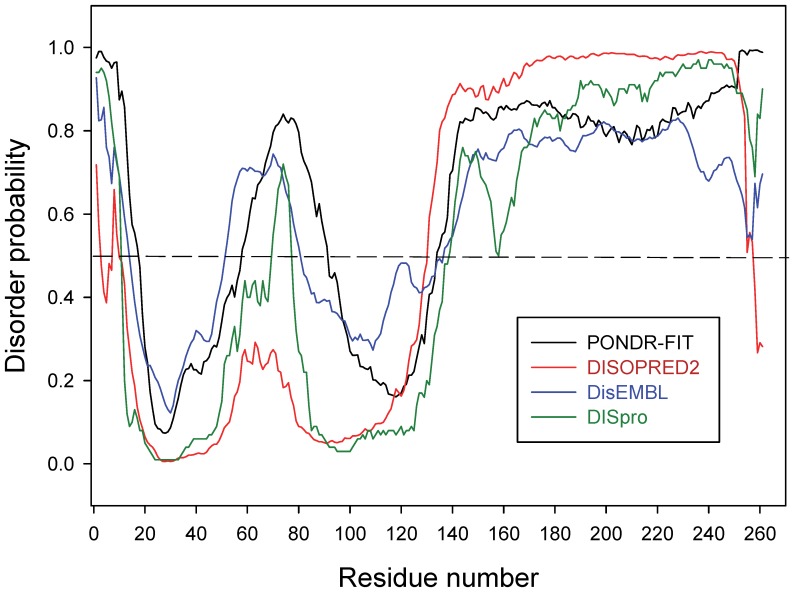
Prediction of PhaF disordered regions. Programs used were PONDR-FIT, DISOPRED2, DisEMBL AND DISpro (see Materials and Methods).

As neither domain is predicted to establish supersecondary or tertiary structure contacts, nor interdomain interactions, the separate modeled domains were simply joined by ligating the backbone of residues 142 and 143, giving rise to the structure shown in Fig. 1E. The result is an extended polypeptide composed of secondary structure segments lacking a defined hydrophobic core. As stated above, this lack of stabilizing contacts might give rise to a partially unfolded structure in the absence of PHA and DNA. To check this, PhaF disorder was predicted by four methods as described in Materials and Methods. Results are shown in Fig. 2. All methods are coincident in pointing at the C-terminal domain as mostly disordered, whereas they also predict an appreciable flexibility in the N-terminal helical moiety of the protein. According to the PONDR-FIT procedure, stretches with more than 50% probability of being disordered in solution comprise residues 1–17, 58–91 and 134–261 (Fig. 2).

Our next step was trying to validate experimentally the most noteworthy features drawn from the model.

### Analytical ultracentrifugation

**Figure 3:**
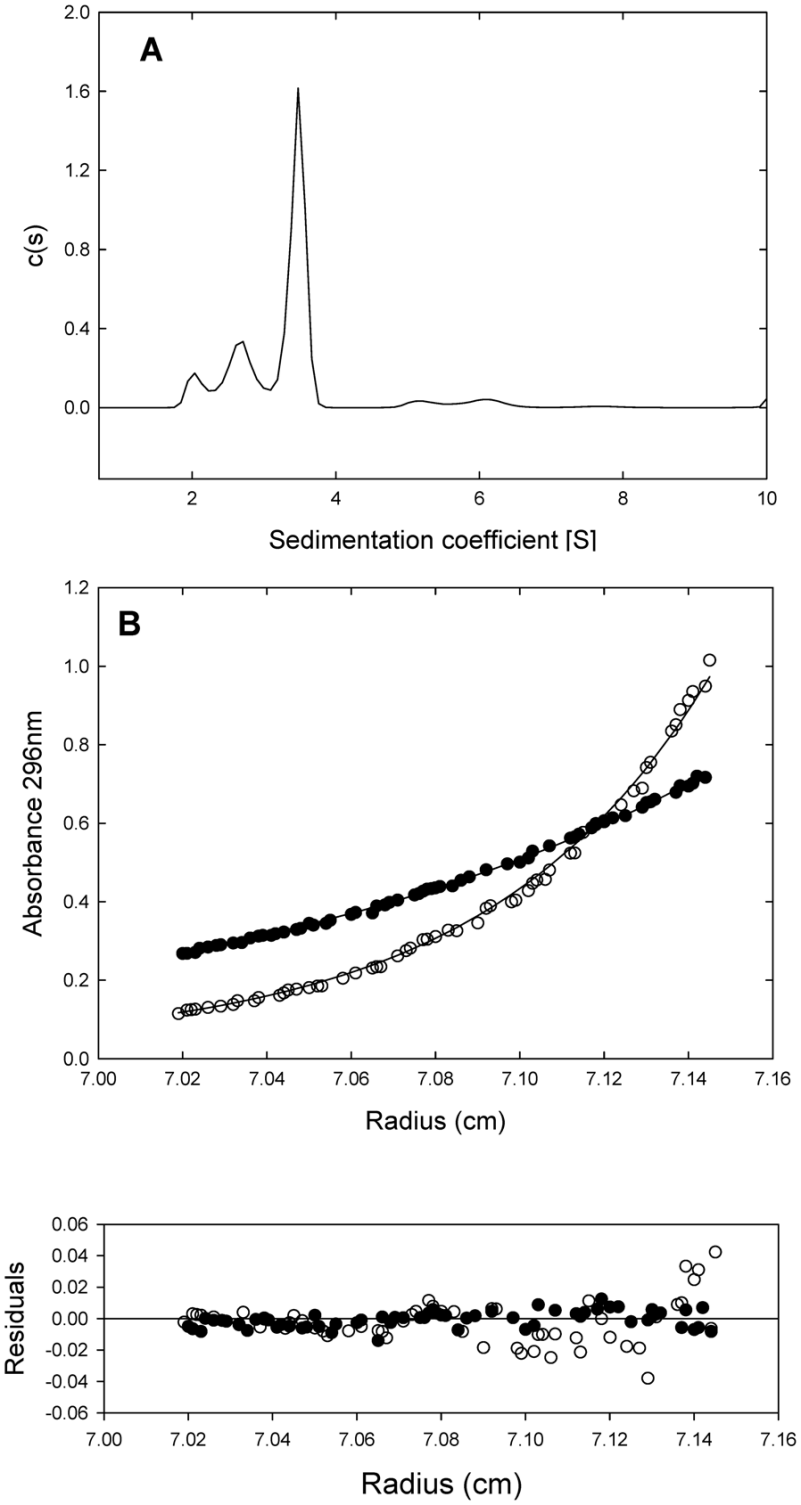
Analytical centrifugation experiments. (**A**) Sedimentation coefficient distributions *c*(*s*) corresponding to the sedimentation velocity of PhaF. (**B**) Sedimentation equilibrium analysis of the association state of PhaF. Sedimentation equilibrium absorbance gradients were carried out at 10,000 r.p.m. (open circles) and 16,000 r.p.m. (closed circles). The solid lines show the corresponding best-fit gradients for a single sedimenting species at equilibrium with a solution average molar mass of 85000 Da. The residuals (difference between the experimental data and the fitted data for each point) are shown at the bottom of this panel.

To check whether PhaF is an oligomer in solution, possibly *via* the predicted leucine-zipper region, a sedimentation velocity experiment was carried out (Fig. 3A) with the phasin purified by hydrophobic chromatography as previously described [18] (Fig. S1). A major species with sedimentation coefficient of 3.4 ± 0.1 S and a frictional ratio (*f*/*f*_o_) of 2.3 (indicative of a highly elongated species) was detected, accounting for around 71% of the loading signal. This species is compatible with an extended tetramer. Two other species with s-values 2.7 ± 0.1 (21%) and 2.0 ± 0.1 S (7%) were also found. The sedimentation coefficient distribution and relative abundance of the species did not change with protein concentration (from 3.8 to 38 µM; data not shown), suggesting that protein exists as a mixture of species at slow equilibrium or as an irreversible non-equilibrium mixture. These results were confirmed by low-speed sedimentation equilibrium experiments done using short solution columns to yield relatively shallow gradients (Fig. 3B). Under these conditions the apparent molar mass becomes essentially independent of the radial distance and is well described by the solution-average molar mass value. In our case, the experimental gradients were best described by an average mass value of 85000 ± 3000 Da, somehow lower than the expected for a tetramer (105000 Da) and compatible with the relative abundance of species previously estimated from the sedimentation velocity data, being the tetramer the predominant one.

### Circular dichroism studies

**Figure 4:**
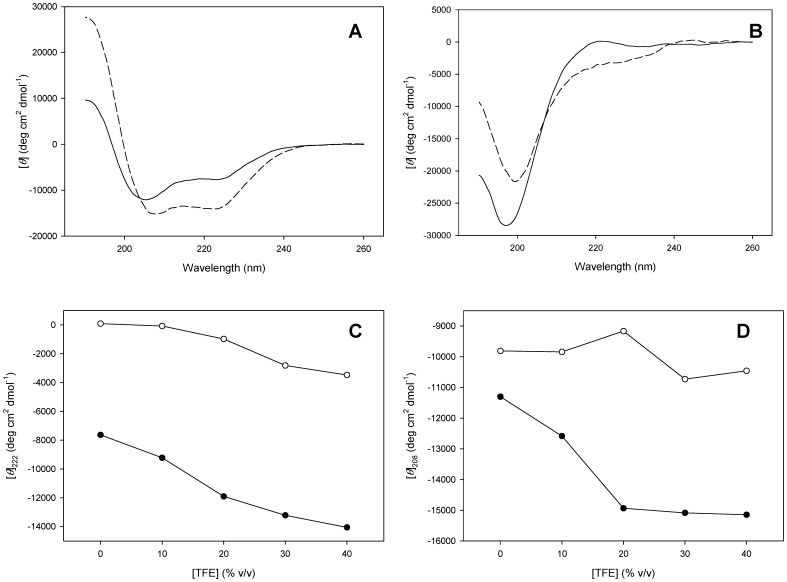
TFE titrations of PhaF and C-PhaF. (**A**) Far-UV CD spectra of PhaF in the absence (solid line) and presence (dashed line) of 40% TFE. (**B**) Far-UV CD spectra of C-PhaF in the absence (solid line) and presence (dashed line) of 40% TFE. (**C**) CD-monitored TFE titration of PhaF (closed circles) and C-PhaF (open circles) following the ellipticity signal at 222 nm. (**D**), same as in (C) but following the signal at 208 nm.

We used circular dichroism as structural probe to determine the thermal stability of PhaF. The far-UV CD spectrum registered at 20 °C displays an appreciable contribution of α-helical structure, with a minimum centered at 206 nm and a shoulder around 222 nm [50] (Fig. 4A), Three different mathematical procedures were employed to deconvolute the far-UV CD spectrum into secondary structure contributions (Table 1). Overall, the methods are coincident in the quantification of α-helix as the predominant structure (around 30%), although with somehow lower values that those predicted from the structural model (44%, Fig. 1). One possible explanation is that, in the absence of other stabilizing contacts with its PHA substrate, the long, N-terminal helix may be partially unfolded in solution, as it is predicted by several disorder prediction methods (Fig. 2), so that it would only acquire its full structure upon binding to the bioplastic granule. In agreement with this hypothesis, the consideration of sequences 1–17 and 58–91 as disordered instead of α-helical, according to the PONDR-FIT program (Fig. 2) reduces the helix content of unligated PhaF down to 31%, in full concordance now with the experimental CD data (Table 1). It would be desirable to analyze the conformational changes induced in the protein by the PHA polymer. However, PHA forms insoluble granules that, at best, may only be prepared as latex emulsions that are not suitable for fluorescence or CD spectroscopy techniques. Instead, we checked the effect of sodium oleate, a common coating component for PHA preparations *in vitro*, as a hydrophobic mimic of PHA [51]. Fig. S3 shows that addition of 1 mM oleate effectively enhances the negative ellipticity of PhaF, with more prominent minima at 208 nm and 222 nm as a consequence of the increase in α-helical content at the expenses of remainder conformations, according to a CDSTTR deconvolution of the spectrum (39% α-helix, 12% β-structures, 49% remainder structures). This suggests that a hydrophobic environment such as that provided by oleate or PHA may interact with the apolar face of the amphipathic N-terminal helix, producing a stabilization effect.

**Table 1:** Secondary structure quantitation of PhaF based in CD data.

Sample	Method	α-Helix (%)	β-Structures (%)	Remainder (%)
PhaF	CDNN	32	18	51
	CONTIN	29	7	64
	CDSSTR	32	11	57
PhaF (40% TFE)	CDNN	42	12	46
	CONTIN	47	7	46
	CDSSTR	55	18	27
C-PhaF	CDNN	N.D.^a^	N.D.	N.D.
	CONTIN	1	4	95
	CDSSTR	1	13	86
C-PhaF (40% TFE)	CDNN	N.D.	N.D.	N.D.
	CONTIN	9	7	84
	CDSSTR	5	8	87
PhaF (whole protein)	Model	44	0	57
	Model (accounting for disorder)^b^	31	0	69

Estimations using the CDNN, CONTIN and CDSSTR procedures, and comparison with that predicted from the three-dimensional model.

a N.D.: Not determined (poor fitting)

b Calculations assuming that the helical-predicted stretches 13–17 and 58–91 are disordered in solution in the unligated protein (according to PONDR-FIT procedure).

With the aim of studying the structure of the N- and C-terminal domains separately, we overexpressed both moieties independently. However, while the C-terminal domain (C-PhaF protein) could be efficiently purified in high amounts [18] (Fig. S1), attempts to obtain the isolated N-terminal domain were unsuccessful due to the high insolubility of the polypeptide. In any case, the far-UV CD spectrum of the C-PhaF domain displays as the most remarkable feature the presence of a single minimum centered at 198 nm (Fig. 4B), which is indicative of random-coil conformation as the major structural component [50]. This is apparently at odds with the predicted superhelical structure of this part of the protein in the model when bound to DNA (Fig. 1E, Fig. S2). Nevertheless, the high number of positively charged lysines in the C-terminal domain (Fig. 1C) is likely to promote strongly repulsive interactions that, unless compensated with its polyanionic DNA ligand, would promote extensive unfolding of the polypeptide in this region. In this sense, this lack of structure does not prevent the isolated C-PhaF polypeptide to recognize and bind its DNA target [18]. Fig. S3 also shows that binding of a DNA oligonucleotide to C-PhaF induces a conformational change in the protein that is reflected in a slight change in its far-UV CD spectrum. The difference spectrum between the free and bound conformations (inset) displays a maximum at 219 nm and a sharp minimum at 194 nm that is compatible with a decreased content in poly(proline)-II structure upon binding [52].

To overcome, insofar as it is possible, the tendency to disorder and therefore to evaluate the secondary structure propensity of the protein, we subjected both the PhaF and C-PhaF proteins to 2,2,2-trifluoroethanol (TFE) titrations, monitoring the CD signal. TFE is a commonly used cosolvent that usually unveils the intrinsic conformational preferences of peptides and proteins [53]. Fig. 4 shows that addition of TFE induces a cooperative conformational change that substantially increases the negative ellipticity leading to minima at 208 nm and 222 nm. Deconvolution of the spectrum recordered at the end of the titration (40% TFE) reveals an increase in α-helical content up to approximately 47%, which is very close to the values predicted from the structural model (44%) (Table 1). On the other hand, titration of C-PhaF induces a much lower change in ellipticity, which is translated to a minor increase in helicity (around 7%, Table 1). This result shows that the tendency of the C-terminal domain of the protein to acquire a regular secondary structure is low even under strong helix-inducing conditions

### Thermal stability

**Figure 5:**
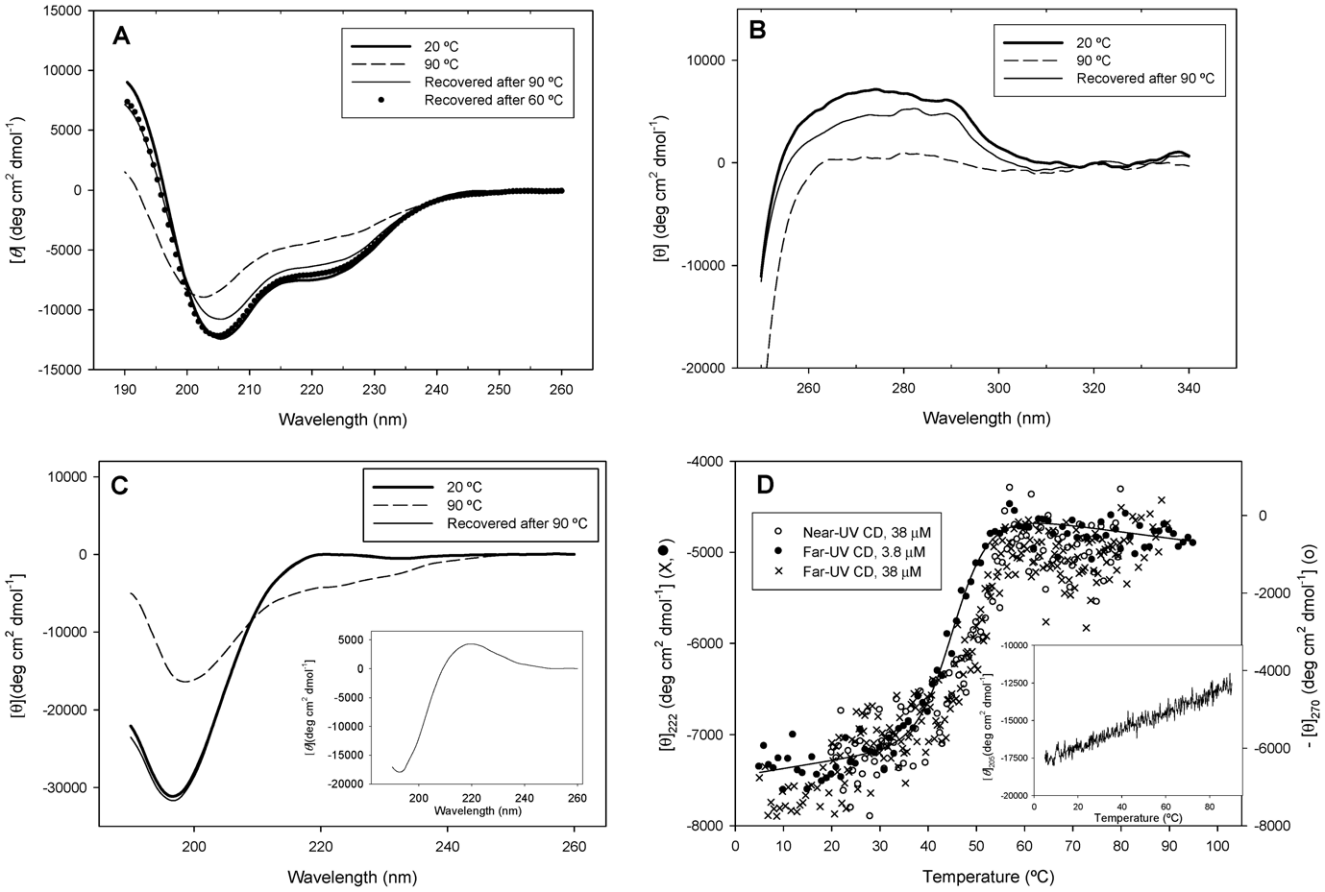
Thermal stability of PhaF. (**A**) Far-UV CD spectra of PhaF at different temperatures. Recovered spectra were registered upon immediate cooling down the scanned sample and after a 30 min waiting period at 20 °C. (**B**) Near-UV CD spectra of PhaF. (**C**) Far-UV CD spectra of the isolated C-terminal domain of PhaF (C-PhaF protein). ***Inset***, difference CD spectrum of C-PhaF (20°C–90 °C). (**D**) CD-monitored temperature scans of PhaF. Solid line indicates fitting of the far-UV CD-monitored transition (protein concentration: 3.8 µM) to the Gibbs-Helmholtz equation (see Materials and Methods). ***Inset***, temperature scan of C-PhaF monitored at 205 nm.

To get an estimation of the stability of the PhaF protein, we performed CD-monitored thermal scans on the full-length PhaF. Incubation up to 90 °C caused extensive unfolding of the protein, as deduced from the decrease in the far-UV CD signal together with the emergence of a single minimum at 202 nm (Fig. 5A). On the other hand, the near-UV CD spectrum indicates the existence of rigid surroundings around aromatic residues (Fig. 5B), probably Trp-88 and Phe-95, which are very close to the leucine-zipper sequence (111–133) and are likely to be affected by the tetramerization process. Heating also caused the disappearance of this near-UV CD signal (Fig. 5B). Both far- and near-UV CD signals underwent a cooperative change with a transition midpoint centered at about 51 °C using a protein concentration of 38 µM (Fig. 5D, Table 2), suggesting that the protein contains a degree of packing in the N-terminal domain (since the C-terminal moiety does not display any cooperative transition – see Fig. 5D, inset). Moreover, Fig. 5D and Table 2 also show that the transition is shifted to lower temperatures as the protein concentration is decreased 10-fold, indicating that the thermal denaturation involves a change in molarity. This, together with the coincidence of the far-UV and near-UV CD scans using the same protein concentration (Fig. 5D), suggests a two-state unfolding-dissociation coupled transition in which the predominant protein tetramer leads to unfolded monomers upon heating, without the accumulation of intermediate species, as indicated in Eq. 2. The hydrophobic core would therefore be conformed by, at least, the packed leucine zipper sequences. On the other hand, the transitions were not fully reversible after sample incubation at 90 °C (Figs. 5A and 5B), but the recovery of the signal substantially improved when the samples were cooled down right after the thermal transition was completed (60 °C) (Fig 5A). This indicates that any irreversible step takes place only once the protein is fully unfolded, so that the equilibrium shown in Eq. 2 is not affected by these post-transitional events and therefore can be analyzed by equilibrium thermodynamics. This is a particular case of the Lumry-Eyring model [54] and has been extensively applied to the analysis of the irreversible unfolding of proteins similar to our case [55], [56]. To ensure this, we carried out a temperature scan at half the heating rate (0.5 K min^−1^) and found very little difference with that acquired at 1 K min^−1^ (Fig S4) and yielding similar thermodynamic parameters (Table 2, see below). This demonstrates that any kinetic effect on the thermal denaturation of the protein is negligible. It is noteworthy that, at the slower heating rate, there occurrs an appreciable loss of ellipticity signal starting at about 72 °C, which was not detected at the faster heating rate (Fig S4). This reinforce our idea that incubation of the protein at high temperatures causes aggregation and/or other irreversible changes in a relatively slow time scale.

**Table 2:** Thermodynamic quantities of the thermal denaturation of PhaF^a^.

Probe	Monomer concentration (µM)	*t*_m_ (°C)	Δ*H*_m_ (kcal/mol)	Δ*G*_int_^o^(kcal/mol)b,c	Δ*G*_int_^o^ (kcal/mol)b,d
Far-UV CD	3.8	45.5±0.2	53.8±2.3	25.0±0.3	23.5±0.3
Far-UV CD	3.8^e^	46.1±0.4	44.2±2.8	24.0±0.6	22.5±0.6
Far-UV CD	38.0	50.8±0.3	103.6±13.5	28.1±1.8	26.2±1.8
Near-UV CD	38.0	51.5±0.3	95.4±12.4	27.3±1.6	25.4±1.6

a Heating rate: 1 K min^−1^ unless otherwise stated

b Data calculated for a temperature of 10 °C

c Assuming Δ*C*_p_ = 0 kcal mol^−1^ K^−1^

d Assuming Δ*C*_p_ = 0.7 kcal mol^−1^ K^−1^

e Heating rate: 0.5 K min^−1^

According to the analytical ultracentrifugation experiments shown above, the minor species that accompany the predominant tetramer are not in fast equilibrium among them, so they should contribute very little to the equilibrium thermodynamic analysis. Table 2 displays the thermodynamic quantities of the thermal transitions as analyzed by the Gibbs-Helmholtz equation (Eqs 3–5). Since Δ*C*_p_ is unknown for PhaF, we tried to obtain its value from the slope of the plot of Δ*H*_m_ versus *T*_m_ from thermograms recorded at different pH's using the procedure described by Swint and Robertson [57]. However, the protein was greatly destabilized on decreasing pH (see below), with very short pre-transitional baselines, so that curve fittings yielded poor results (data not shown). Alternatively, we employed two estimates of Δ*C*_p_. The extended nature of the predicted structure of PhaF points to a predictably low Δ*C*_p_ value, directly related to the increment in accesible surface area upon unfolding [58]. We firstly estimated for our calculations a value of 0.7 kcal mol^−1^ K^−1^, that has been determined experimentally for the designed 31-aa AB_SS_ leucine zipper [59] (the predicted leucine zipper sequence of PhaF spans 22 residues – see Fig. 1A). Nevertheless, even lower values have been reported for other coiled-coils [60]. In any case, even estimating Δ*C*_p_ = 0 kcal mol^−1^ K^−1^, the calculated free energies of unfolding, extrapolated to 10 °C, barely differ in 10%, that lies within the experimental error (Table 2).

A thermal stability analysis of the isolated C-PhaF was carried out by monitoring the effect of the temperature on its far-UV CD spectrum. Fig. 5C shows that at high temperature (90 °C) the intensity of the spectrum substantially decreases, a change that is reversible upon cooling down the sample. While some negative ellipticity also appears in the 215–235 nm range at 90 °C, this should not be necessarily taken as indicative of residual structure, since Privalov *et al.* [61] noticed similar changes in completely unfolded peptides and ascribed this phenomenon to non-specific effects of temperature on the CD spectrum. An alternative/additional explanation to this phenomenon arises from the analysis of the difference spectrum of C-PhaF between 20°C and 90°C (a strong minimum at 193 nm and a maximum at 220 nm, Fig. 5C inset), which suggests a loss of poly(proline)-II structure upon denaturation [52]. In any case, Fig 5D (inset) displays a non-cooperative loss of CD signal with increasing temperature, which is typical of peptides devoid of fixed tertiary structure, thus demonstrating the absence of a significant hydrophobic core and suggesting that the C-terminal domain of PhaF is essentially unfolded in the absence of its DNA ligand.

### Chemical unfolding

**Figure 6:**
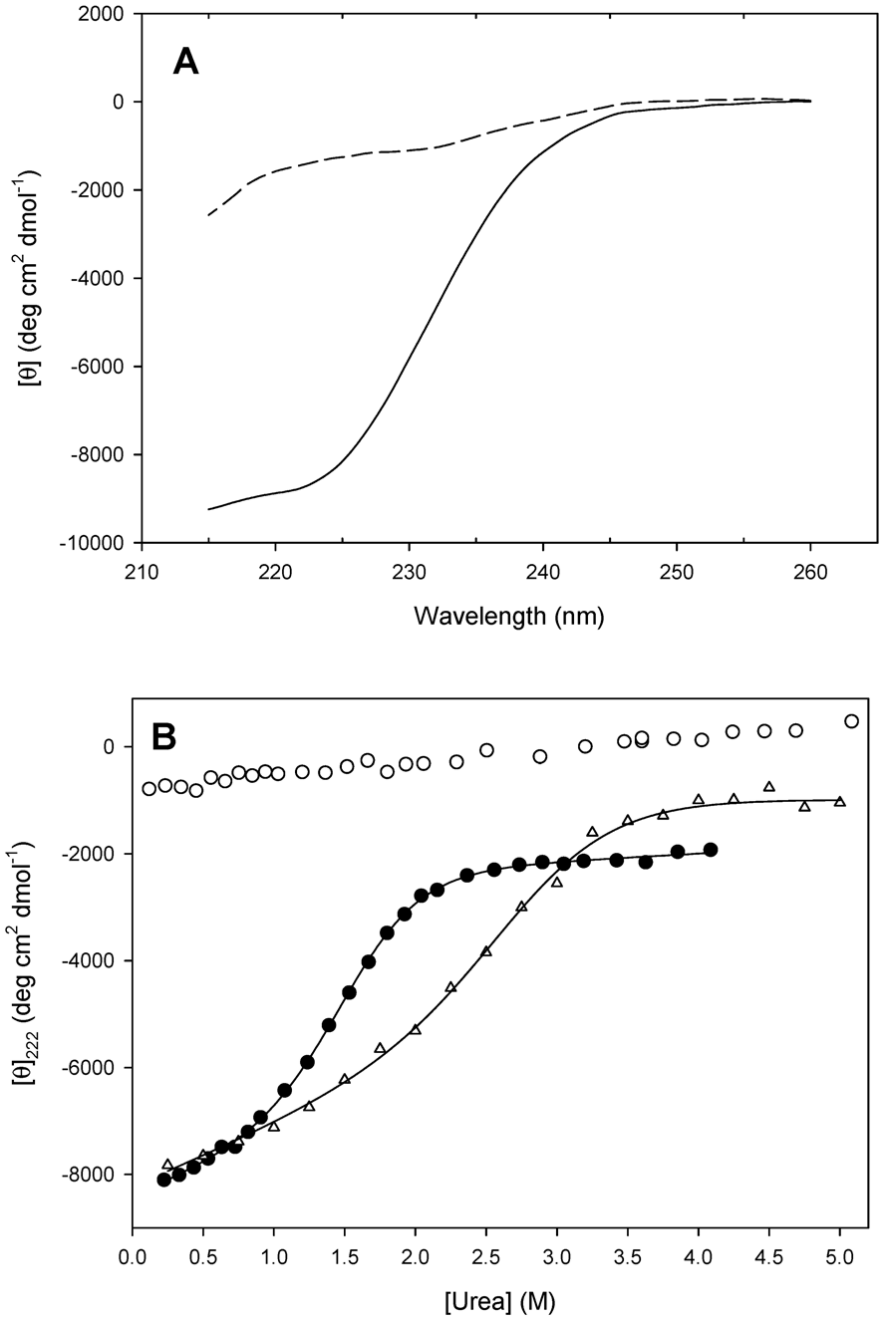
Chemical stability of PhaF. (**A**) Far-UV CD spectra of PhaF in the absence (solid line) and the presence (dashed line) of 4.1 M urea. (**B)**. CD-monitored urea titration of 3.8 µM PhaF (closed circles), 11.4 µM PhaF (open triangles) and C-PhaF (open circles). The solid lines represents the fitting to a two-state equilibrium denaturation transition (see text).

PhaF (3.8 µM, total monomer concentration) was subjected to equilibrium denaturation by urea monitored by far-UV CD. Experiments were carried out at low temperature (10 °C) in order to acquire reliable baselines since the stability of the protein is only marginal at room temperature (Fig. 5D). As shown in Fig. 6, the protein was fully unfolded in a cooperative way using relatively low concentrations of denaturant even at 10 °C. The thermodynamics of the transition was analyzed by the linear extrapolation method (Eqs. 5 and 6), yielding the following fitting parameters: *m* = 2.1 ± 0.1 kcal mol^−1^ M^−1^; [urea]_½_ = 1.5 ± 0.1 M; Δ*G*_eff_^o^ = 3.2 ± 0.2 kcal mol^−1^; and Δ*G*_int_^o^ = 22.2 ± 0.2 kcal mol^−1^. This value is in good agreement with those calculated from the thermal unfolding experiments (Table 2). When the protein concentration increased 3-fold (11.4 µM, total monomer concentration), the urea midpoint also shifted to higher concentrations (Fig. 6), showing again a dependence on protein concentration in agreement with the change in molarity upon unfolding. In this case, the thermodynamic parameters were: *m* = 1.4 ± 0.1 kcal mol^−1^ M^−1^; [urea]_½_ = 2.7 ± 0.1 M; Δ*G*_eff_^o^ = 3.8 ± 0.2 kcal mol^−1^; and Δ*G*_int_^o^ = 21.0 ± 0.2 kcal mol^−1^. Finally, lack of structure in the C-terminal region of the protein was confirmed by the linear, non-cooperative change in CD signal of the C-PhaF polypeptide (Fig. 6B).

### pH stability

**Figure 7:**
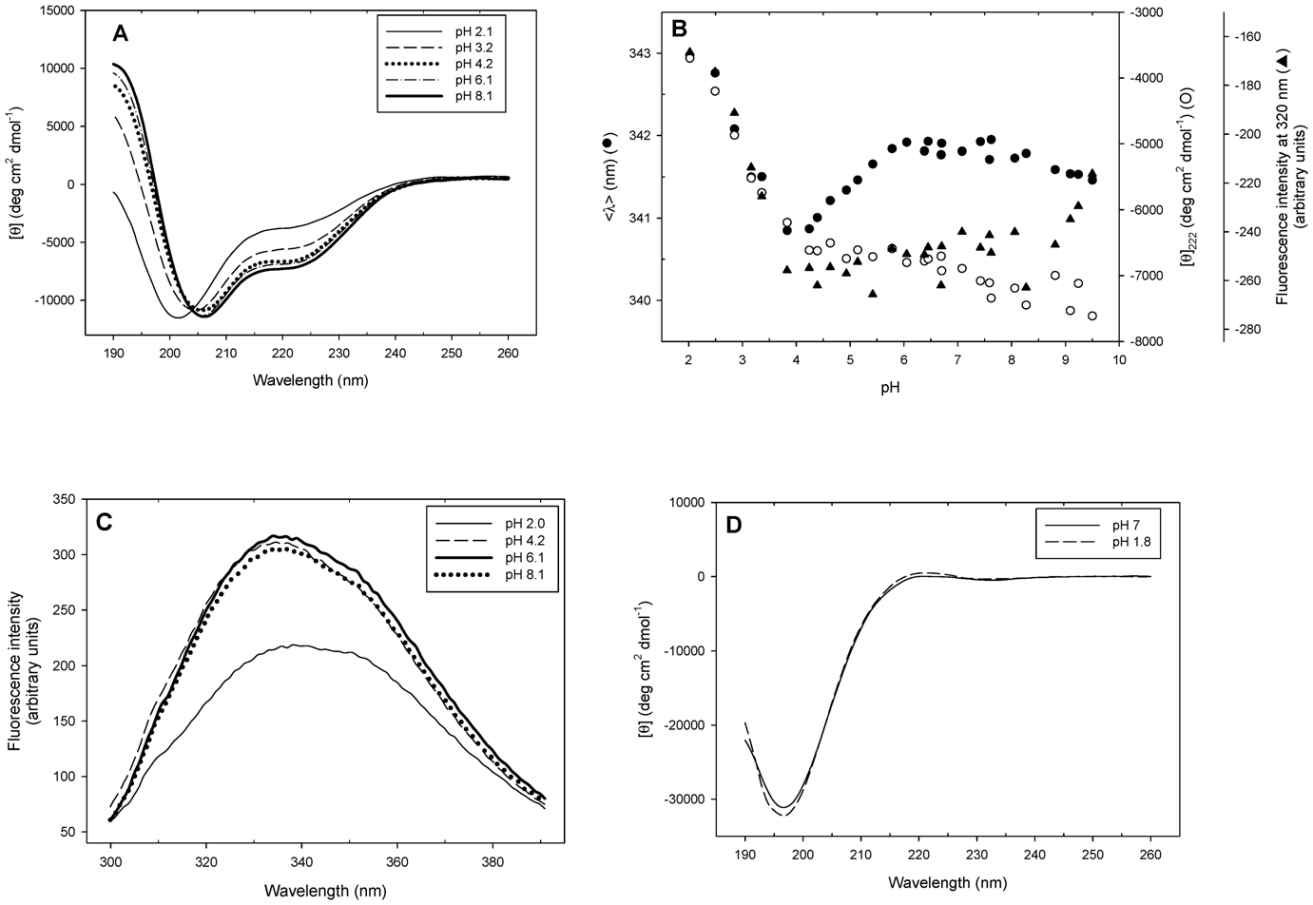
pH stability of PhaF. (**A**) Far-UV CD spectra of PhaF registered at different pH's. (**B**) pH titration of PhaF monitored by far-UV CD (open circles), average tryptophan fluorescence emission intensity (closed circles) and fluorescence intensity at 320 nm (triangles). (**C**) Intrinsic tryptophan fluorescence spectra of PhaF at different pH's. (**D**) Far-UV CD spectra of C-PhaF.

The structural integrity of PhaF was assessed at different pH's by far-UV CD and intrinsic fluorescence spectroscopies (Figs. 7 A–C). Fig. 7A shows the reduction of the 206 nm and 222 nm minima and the appearance of a band at 200 nm upon decreasing the pH, reflecting the loss of α-helical structure in favour of random-coil conformations. The existence of an isodichroic point at 204 nm points to a two-state transition, without detectable intermediates. Plotting the far-UV CD signal *vs* pH shows a low-intensity, linear change in CD signal between pH 9.5 and 4.0, and an incomplete transition starting below pH 4.0 (Fig. 7B). This indicates that the protonation of one or more aspartate or glutamate side chains in the N-terminal domain of PhaF is responsible for the pH stability of the phasin. On the other hand, the tryptophan fluorescence spectrum (Fig. 7C) displays a maximum at 336 nm that is shifted at lower pH's to higher wavelengths (343 nm), concomitantly with a decrease in fluorescence intensity. This is in accordance with tryptophan exposure to the solvent arising from protein unfolding. Upon examining the change on the average emission intensity (<λ>) (Fig. 7B), the low-pH CD-monitored transition was reproduced, but additionally a second transition appeared centered at pH 5.2. Nevertheless, since no significant changes were observed in this pH range in terms of secondary structure (Fig. 7A) and overall fluorescence intensity (Fig. 7B and 7C), we believe that this additional transition arises from the protonation of some acidic group(s) that locally affect the polarity of the environment around the tryptophan residues, but without inducing any appreciable conformational change in the protein. In fact, plotting the raw fluorescence intensity only yields a single transition similar to the CD-monitored one (Fig. 7B). Finally, as all acidic residues of PhaF except one (Glu-190), are located in the N-terminal domain, it should be expected that the pH-induced conformational changes were also restricted to this region of the protein: lack of CD spectral changes in the C-PhaF protein at different pH's (Fig. 7D) supports this hypothesis.

## Discussion

The traditional concept or proteins as rigid, compact entities, the flexibility of which is limited to moderate conformational rearrangements necessary to carry out any molecular recognition events, is being currently challenged with the increasing description of polypeptides lacking defined secondary and tertiary structures, the so-called intrinsically disordered proteins [62]. Far from being curious exceptions, intrinsic disorder is a widespread mechanism allowing a polypeptide to suitably adapt itself to its biological role, and to carry out very important physiological functions including regulation, signalling, chaperoning and nucleic acid binding [63], [64].

Phasins interact with a hydrophobic, lipid-coated insoluble substrate (the polyhydroxyalkanoate intracellular granule), providing it with a surface layer that avoids granule coalescence. Other functions proposed for phasins are: controlling the number and surface of granules, protecting the host cell by contributing to coverage of the hydrophobic surface of the polymer, preventing protein misfolding on the hydrophobic granule [3] or serving as a storage source of nitrogen [14]. In many occasions, stabilization of the PHA granule must simultaneously coincide with other biological functions. In the case of PhaF, it has been demonstrated that this phasin binds DNA non-specifically and plays an essential role in cytoplasmic granule localization and segregation into daughter cells upon cell division [18]. Such different functions can only be supported by a peculiar PhaF structure distributed in separated domains that recognize their different macromolecular substrates (PHA and DNA). According to their structural characteristics, phasins are good candidates for becoming part of the intrinsically disordered protein family [62], as their mean hydrophobicity is low (reducing the possibility of an appreciable hydrophobic core) and there is a high net charge repulsion, especially in the C-terminal domain (Fig. 1C; Fig. S2). Moreover, their regulatory biological role demands a high degree of plasticity to simultaneously adjust to the structure of such macromolecules as chromosomal DNA and PHA granules.

Given the specific bifunctionality of PhaF not previously found in other proteins, it is hardly surprising that no homologs have been found yet in the databases of proteins with known 3-D structure, from which a structural model of PhaF could be derived. However, the neat separation into domains that can be discerned from the inspection of its sequence [17] and experimental evidence [18], [27], [28] (see Introduction) prompted us to model such domains separately, and subsequently link the resulting structures. Then, the model was subjected to experimental validation using hydrodynamic and spectroscopical techniques. Fig. 1E displays the final structural model of a PhaF monomer, based in secondary structure predictions (N-terminal domain and C-terminal tail, Figs. 1A and D) and sequence similarity with the AlgP regulator (C-terminal domain, Fig. 1C). According to our model, the protein has an extended shape, in which the N-terminal, PHA-binding domain maintains little, if any, interaction with the C-terminal, DNA-binding moiety. This separation of domains ensures that the bifunctionality of the protein is accomplished without any spatial hindrance.

The N-terminal domain is predicted to contain a long, amphipathic α-helix (Fig. S2) which is partially disordered in the absence of PHA (Fig. 2), although this partial unfolding may be reverted in the presence of hydrophobic PHA mimics such as sodium oleate (Fig. S3) or structure-stabilizing cosolvents such as TFE (Fig. 4A). A disorder-to-order transition upon ligand binding is a common characteristic of intrinsically disoredered proteins [62]. The long N-terminal helical stretch is followed by a short coiled-coil sequence, possibly a leucine zipper (Figs. 1A and B), that is be involved in the tetramerization of the protein as unveiled by the ultracentrifugation experiments (Fig. 3). Circular dichroism spectroscopy provides experimental support for the helical nature of the N-terminal domain (Fig 4A, Table 1). Moreover, the dependence of the thermal and chemical stability on protein concentration (Figs. 5D and 6B, Table 2) is in agreement with the oligomeric nature of PhaF. We have not detected in the literature any references so far to coiled-coil sequences in polyhydroxyalkanoate-associated proteins, so that, to the best of our knowledge, this might be the first documented case of such an oligomerization motif in phasins. In order to check the distribution of coiled-coil sequences among other polyhydroxyalkanoate-associated proteins, we analyzed a total of 973 sequences belonging to the UniProtKB database containing the term "phasin". The COILS utility identified 499 sequences (51.3% of the database) with a probability higher than 75% of containing coiled-coil stretches (Table S1), plus an additional 188 sequences with a probability between 25% and 75% (19.4% of the database, Table S2). The best hits encompass a wide variety of organisms and gene lengths. The fact that approximately 70% of the analyzed phasin sequences show hints of coiled-coils to an appreciable degree is remarkable and points to this motif as a widespread mechanism for the oligomerization of PHA-associated proteins. It is noteworthy that the accompanying PhaI phasin in *P. putida* KT2440 holds a predicted coiled-coil sequence within its primary structure as well (Table S1).

With respect to the C-terminal moiety of the protein, the model assumes a superhelical structure based on that of the AlgP regulatory protein (Figs. 1C and 1E; Fig. S2) with basic residues adapted to electrostatically interact with the phosphate backbone of DNA. However, this structure should be only stable upon binding to the nucleic acid, thus overcoming the otherwise strong ionic repulsion between the lysine residues. A coincident prediction by several procedures (Fig. 2) supported by experimental data such as a mostly featureless CD spectra (Fig. 4B), a moderate response to the addition of high concentrations of helix-inducing TFE (Fig. 4B) and the absence of cooperative unfolding transitions induced by heat (Fig. 5D, inset), urea (Fig. 6B) or pH (Fig. 7D) suggest that the C-terminal domain is indeed natively unfolded in solution in the absence of DNA, a fact that nevertheless does not prevent the domain to recognize its polyanionic ligand [18] (Fig. S3). This is a similar case than that of histones, an intrinsically disordered protein family for which its disorder is absolutely essential to carry out their function [65]. With respect to the 35-aa C-terminal tail that follows the DNA-binding moiety of PhaF, it lacks any basic residues and the structure is unequivocally predicted as random coil by all methods tested (Fig. 1D). However, the difference CD spectrum of C-PhaF at 20°C and 90°C (Fig. 5C, inset) suggests that some poly(Pro)-II conformation is present in the protein, a secondary structure not taken into account in most predictors. The 35-aa tail intriguingly contains repeated sequences with the PXXP signature typical of SH3- binding motifs [66] and that has been found within poly(proline)-II helices [67]. Moreover, a FASTA search with this sequence revealed that similar stretches are found in many other polypeptides (data not shown), among them other phasins and related proteins such as the above mentioned AlgP [68], and even the H1 histone. This suggests that, despite this apparent lack of structure, this C-terminal tail must fulfil a relevant biological role that is unknown at the moment.

The intrinsic disorder detected in the C-terminal part of PhaF and in a part of the N-terminal moiety prompted us to check, in the above mentioned UniProtKB database, whether this could be also a characteristic shared by other phasins. When the database was subjected to the PONDR-FIT analysis, we found that a remarkable content of disorder propensity was unveiled in most of the cases (data not shown). In order to set a more formal definition, we arbitrarily selected those sequences displaying more than 50% of the residues with a disorder disposition higher than 0.5 according to PONDR-FIT, and the results are shown in Table S3. Out of 973 sequences, a remarkable 24% (234 sequences) complied with our selective disorder threshold. We therefore hypothesize that an important subset of phasins may constitute a new family of intrinsically disordered proteins not described so far.

Upon inspection of the model, it follows that most of the unligated PhaF stability should be acquired through oligomerization and that the protein mainly acquires its stability from quaternary-, rather than tertiary-structure contacts. In this sense, thermal and chemical denaturations can be treated as tetramer-monomer coupled unfolding-dissociation equilibria, yielding a folding energy of around 25 kcal mol^−1^ (Figs. 5 and 6; Table 2). This value lies within the range of other proteins that oligomerize through coiled coils of the same length, such as the 21-aa Lac21E/K heterotetramer (22.4 kcal mol^−1^) [69].

**Figure 8:**
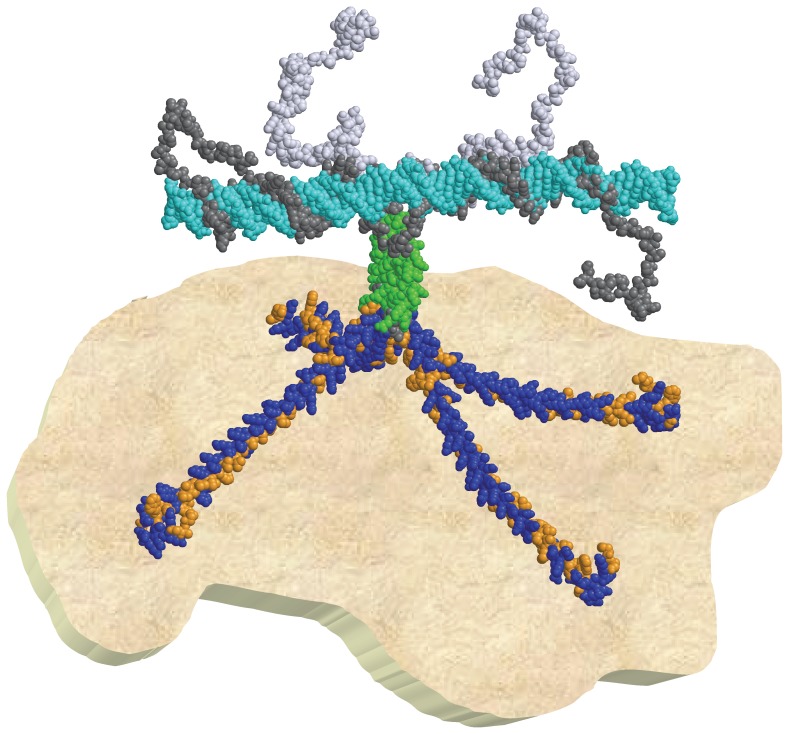
A model of the interaction between PHA granules, phasins and chromosomal DNA in Pseudomonas putida KT2440. A PhaF tetramer is depicted interacting with the PHA granule while also attached to a fragment of nucleoid DNA. The hydrophobic residues in the N-terminal domain are shown in orange, while the polar ones are colored blue. The leucine zipper is colored green, and the DNA is depicted in cyan. Figure was rendered with RASTOP 2.2 (http://www.geneinfinity.org/rastop/) only for visualization purposes, so that no atomic detail is intended.

All our results, together with previous biochemical data [18] allow us to delineate a structural model of the PHA/phasin/DNA biological entity (Fig. 8). In this scheme, the hydrophobic face of the amphipathic N-terminal helixces are associated to the PHA granule, either through its lipid coating or even to the naked polyester itself (unpublished results). The positive interaction unveiled from the conformational change induced in PhaF by PHA mimics such as oleate (Fig. S3) are in accordance with this hypothesis. In turn, the hydrophillic side faces the solvent, thus providing the granule surface a polar character that prevents granule coalescence or non-specific protein association through hydrophobic interactions. On the other hand, the C-terminal domain binds to the chromosomal DNA (and perhaps to cytoskeletal-like proteins) to facilitate the needle-type array granule structure at cellular centre, and linking DNA replication and granule uniform distribution to the daughter cells upon cellular division [18]. Given the presumably low, if any, interdomain interactions, the oligomeric state of PhaF in solution is likely to be conserved when bound to PHA and DNA, providing a multipoint attachment system whose affinity for the macromolecular ligands would be greatly enhanced compared to the monomeric situation thanks to multivalent effects. This could be one of the reasons why the coiled-coil (oligomerization) motif is so widely spread among phasins (Tables S1 and S2). Furthermore, the fact that the PhaI phasin, a companion of PhaF associated to the PHA granule, might also contain a coiled-coil motif, suggests that heterodimers/heterotetramers are also likely to happen, although experimental confirmation of this point must await until suitable amounts of PhaI protein can be purified.

To sum up, we have developed a structural model of the PhaF phasin from *Pseudomonas putida* KT2440 that has been experimentally tested and that may provide a molecular basis for its biological role in PHA granule stabilization, solvation, intracellular arrangement and equal distribution to daughter cells. Our hypothesis contemplates a tetrameric, intrinsically disordered protein in the absence of its ligands PHA and DNA, that acquires a higher degree of secondary structure when interacting with its binding partners. This disorder-order transition is typical of most of the intrinsic disordered proteins described so far [62]. Our results may help to understand the function of these important proteins involved in the metabolism of the PHA bioplastics, and could also be of utility to obtain improved variants of the BioF affinity tag [27], [28]. Moreover, some structural features of PhaF are shared among a high number of other phasins, even from unrelated organisms, namely the conservation of coiled-coil oligomerization sequences and an appreciable level of intrinsic disorder, evidences that deserve further investigations.

## Supporting Information

Figure S1 **Purification of proteins.** (**A**) Purification of PhaF by butyl sepharose. Lanes: M, molecular weight markers (General Electric Healthcare); 1, total extract of *Escherichia coli* BL21(DE3) [pETPhaF]; 2 soluble fraction of the extract; 3, inclusion bodies solubilized and refolded in 20 mM sodium phosphate; 4, flowthrough upon application on a buthyl-sepharose column; 5-7, fractions of protein purified upon elution with 20 mM sodium phosphate pH 7.0. (**B**) Purification of the carboxy-terminal domain of PhaF (C-PhaF) by ionic exchange chromatography. Lanes: M, molecular weight markers; 1, total extract of *E. coli* BL21(DE3) [pETCterm]; 2, total extract from *E. coli* BL21(DE3) harbouring the pET-29a(+) plasmid without insert as a control; 3-5, eluted fractions of purified C-PhaF protein upon elution with 0.7 M Tris-HCl buffer, pH 8.8, plus 4 M NaCl.
(PDF)

Figure S2 **Structural features of the modeled N- and C-terminal domains of PhaF.** (**A**) two spacefill views of the N-terminal moiety, showing polar (blue) and hydrophobic (yellow) residues. (**B**) a detail of the N-terminal domain showing acidic (red) and basic (cyan) side chains. (**C**) two spacefill views of the C-terminal moiety, highlighting the basic side chains in blue.
(PDF)

Figure S3 **Effect of compounds on PhaF structure.** (**A**) Far-UV CD spectra of PhaF in 20 mM sodium phosphate buffer in the absence (solid line) and the presence (dashed line) of 1 mM sodium oleate. (**B**), far-UV CD spectra of C-PhaF in 20 mM sodium phosphate buffer in the absence (solid line) and the presence (dashed line) of 9 mM nspDNA. *Inset*, difference spectrum calculated from those of free and DNA-bound samples.
(PDF)

Figure S4 **Effect of heating rate on PhaF thermal denaturation monitored by far-UV CD.** Heating rate was set to 0.5 K min^−1^ (open circles) and 1 K min^−1^ (closed circles). Solid line indicates fitting of the 0.5 K min^−1^ transition (protein concentration: 3.8 µM) to the Gibbs-Helmholtz equation (see Materials and Methods). Ellipticities above 72 °C were not taken into account for the fitting as they may reflect late aggregation events.
(PDF)

Table S1 **Phasins from the UniProtKB database with >75% probability of containing coiled-coil sequences according to the COILS procedure.**
(XLS)

Table S2 **Phasins from the UniProtKB database with probability of containing coiled-coil sequences between 25%-75% according to the COILS procedure.**
(XLS)

Table S3 **Phasins from the UniProtKB database with intrinsic disorder probability according to the PONDR-FIT procedure.**
(XLS)
