# Supplementary figures and images for: A New Family of Intrinsically Disordered Proteins: Structural Characterization of the Major Phasin PhaF from Pseudomonas putida KT2440
Source: PLoS One. 2013 Feb 15;8(2):e56904. doi: 10.1371/journal.pone.0056904 (PMC3574117; doi:10.1371/journal.pone.0056904)

Supporting Figure S1

A

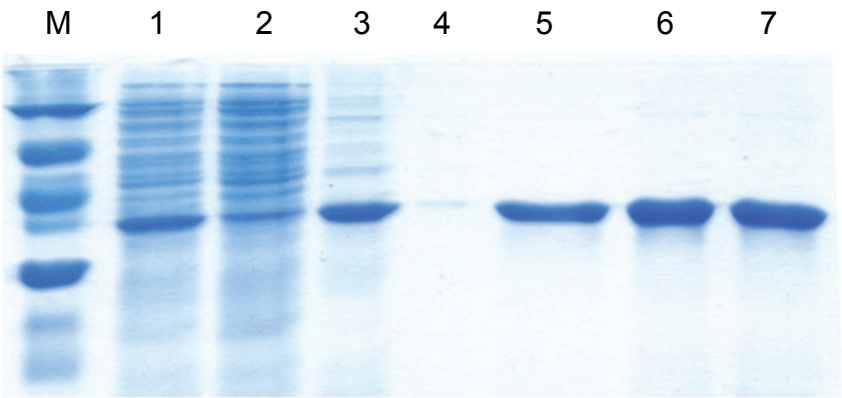

B

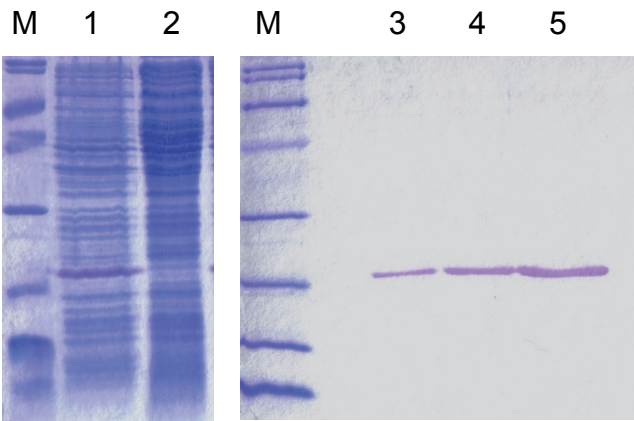

Supplement: Figure S1 — Purification of proteins. (A) Purification of PhaF by butyl sepharose. Lanes: M, molecular weight markers (General Electric Healthcare); 1, total extract of Escherichia coli BL21(DE3) [pETPhaF]; 2 soluble fraction of the extract; 3, inclusion bodies solubilized and refolded in 20 mM sodium phosphate; 4, flowthrough upon application on a buthyl-sepharose column; 5-7, fractions of protein purified upon elution with 20 mM sodium phosphate pH 7.0. (B) Purification of the carboxy-terminal domain of PhaF (C-PhaF) by ionic exchange chromatography. Lanes: M, molecular weight markers; 1, total extract of E. coli BL21(DE3) [pETCterm]; 2, total extract from E. coli BL21(DE3) harbouring the pET-29a(+) plasmid without insert as a control; 3-5, eluted fractions of purified C-PhaF protein upon elution with 0.7 M Tris-HCl buffer, pH 8.8, plus 4 M NaCl. (PDF) [file pone.0056904.s001.pdf]

Supporting Figure S2

A

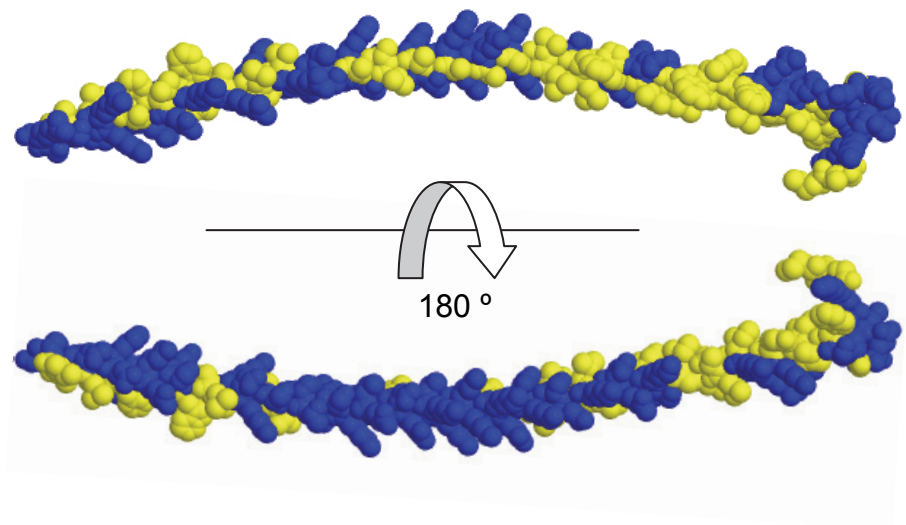

B

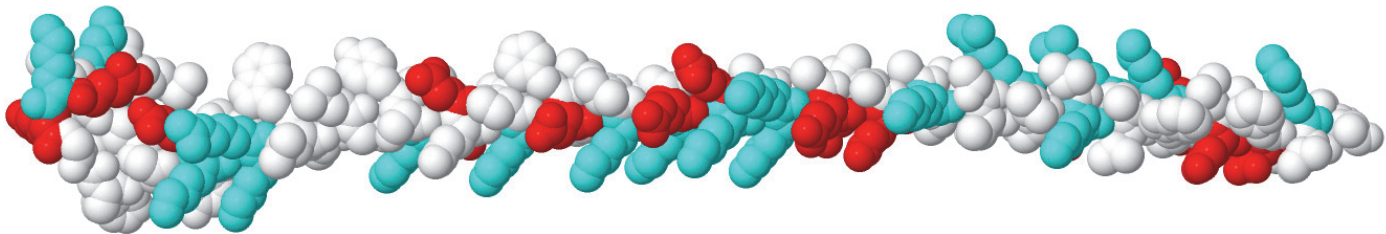

C

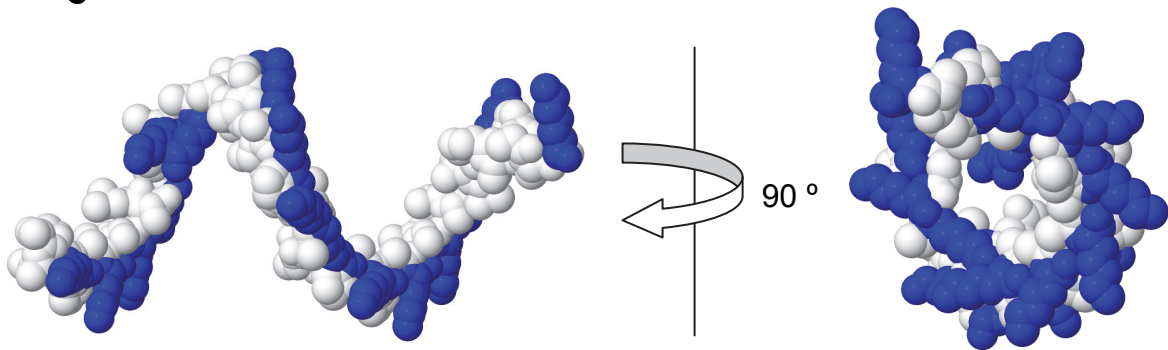

Supplement: Figure S2 — Structural features of the modeled N- and C-terminal domains of PhaF. (A) two spacefill views of the N-terminal moiety, showing polar (blue) and hydrophobic (yellow) residues. (B) a detail of the N-terminal domain showing acidic (red) and basic (cyan) side chains. (C) two spacefill views of the C-terminal moiety, highlighting the basic side chains in blue. (PDF) [file pone.0056904.s002.pdf]

Supporting Figure S3

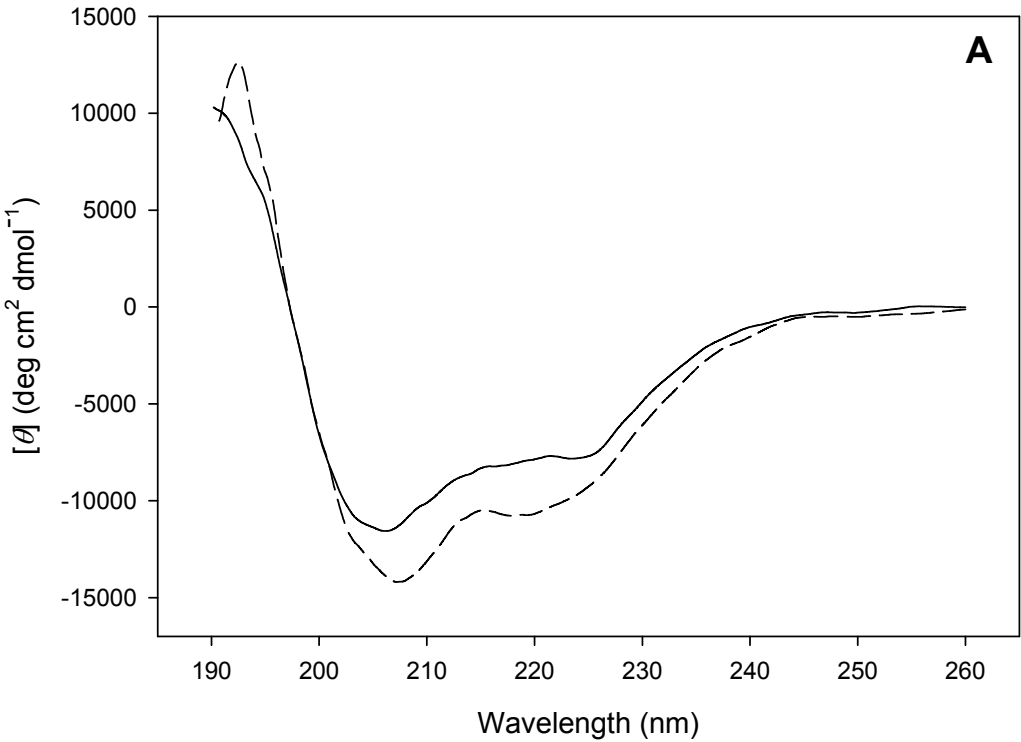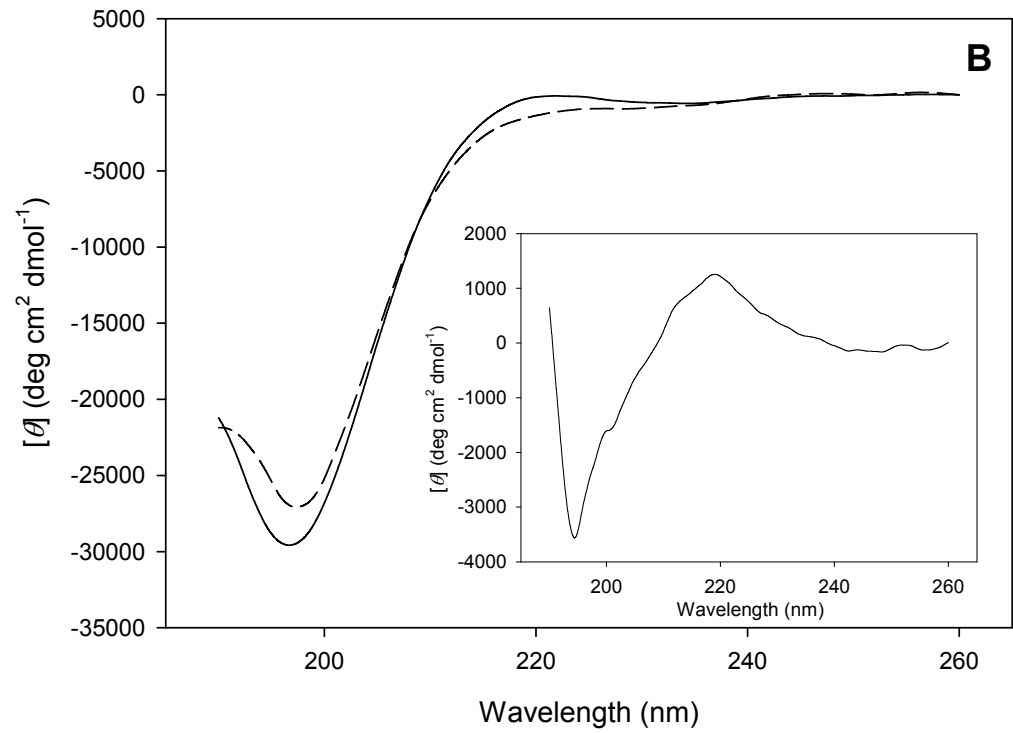

Supplement: Figure S3 — Effect of compounds on PhaF structure. (A) Far-UV CD spectra of PhaF in 20 mM sodium phosphate buffer in the absence (solid line) and the presence (dashed line) of 1 mM sodium oleate. (B), far-UV CD spectra of C-PhaF in 20 mM sodium phosphate buffer in the absence (solid line) and the presence (dashed line) of 9 mM nspDNA. Inset, difference spectrum calculated from those of free and DNA-bound samples. (PDF) [file pone.0056904.s003.pdf]

**Supporting Figure 4**

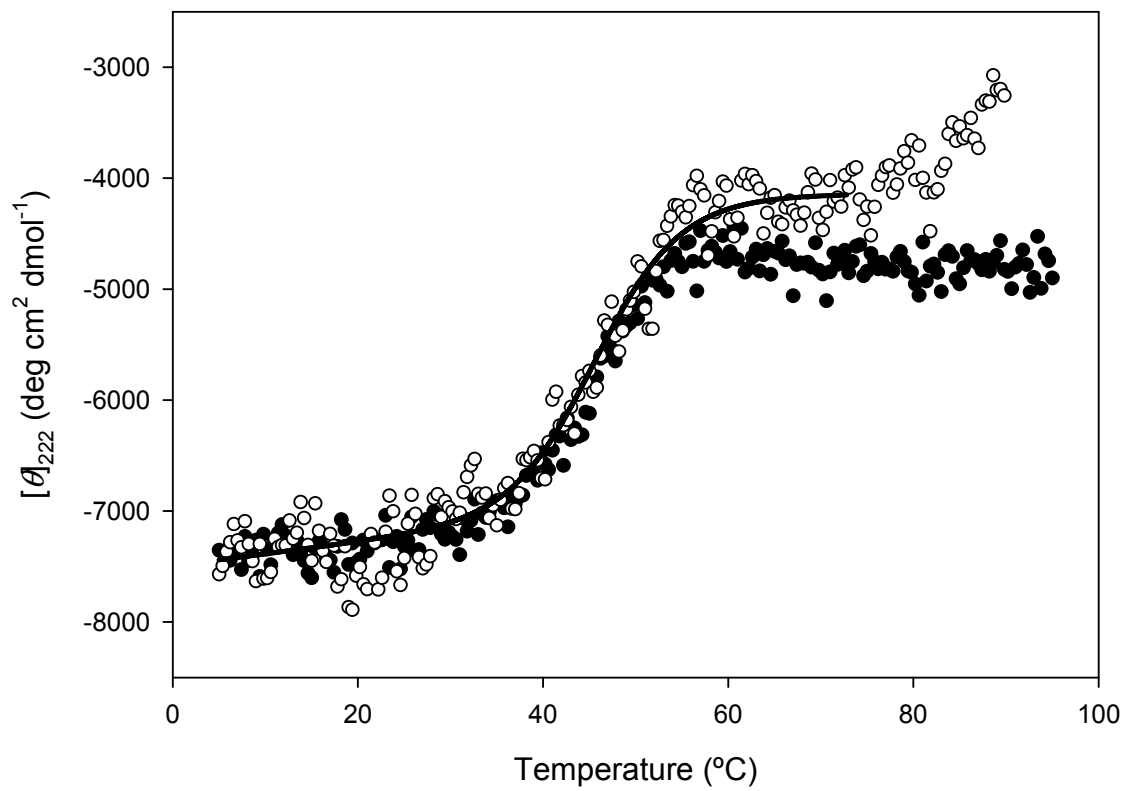

Supplement: Figure S4 — Effect of heating rate on PhaF thermal denaturation monitored by far-UV CD. Heating rate was set to 0.5 K min−1 (open circles) and 1 K min−1 (closed circles). Solid line indicates fitting of the 0.5 K min−1 transition (protein concentration: 3.8 µM) to the Gibbs-Helmholtz equation (see Materials and Methods). Ellipticities above 72 °C were not taken into account for the fitting as they may reflect late aggregation events. (PDF) [file pone.0056904.s004.pdf]
